# Recruitment of pregnant and breastfeeding women in pharmacokinetic studies: strategies, opportunities, barriers, and recommendations

**DOI:** 10.1186/s13104-024-06946-x

**Published:** 2024-10-17

**Authors:** Ritah Nakijoba, Aida N. Kawuma, Simon Peter Asiimwe, Christine Turyahabwe, Jovia Christine Tabwenda, Jacqueline Kyeyune, Johnson Magoola, Francis Williams Ojara, Catriona Waitt

**Affiliations:** 1 Infectious Diseases Institute, Makerere University College of Health Sciences, Kampala, Uganda; 2 Department of Pharmacology and Therapeutics, Gulu University, Gulu, Uganda; 3 Department of Pharmacology and Therapeutics, University of Liverpool, Liverpool, UK

**Keywords:** Tuberculosis, Malaria, Breastmilk, Pharmacokinetics, Lactation, Pregnant and breastfeeding women

## Abstract

Pregnant and breastfeeding women are often under-represented in clinical research, including pharmacokinetic studies, due to ethical and logistical challenges. This paper examines strategies to improve the recruitment and retention of this demographic in pharmacokinetic research, drawing on experiences from five studies conducted at the Infectious Diseases Institute, Makerere University, Uganda. Key strategies implemented include Community Advisory Board meetings, the involvement of Peer Mothers as Co-Investigators, established recruitment sites, the use of safety protocols, and the utilization of diverse communication platforms, including social media and stakeholder meetings. Despite these efforts, substantial barriers, such as scheduling conflicts and frequent staff turnover at recruitment sites, continue to threaten progress. The paper recommends flexible scheduling, strengthening public engagement, and transparent demonstration of adherence to ethical principles; justice, non-maleficence, respect, and beneficence to ensure the safety and inclusivity of pregnant and breastfeeding women. The inclusion of this population in pharmacokinetic studies is essential for providing evidence-based care that meets their unique health needs.

## Introduction

Pregnant and breastfeeding women and their infants are disproportionately under-represented in clinical pharmacology research due to ethical concerns about potential risks [1] fear of potential litigation [2], and the complex physiological changes during pregnancy and lactation that complicate study design and interpretation [3]. Consequently, in many situations, pregnancy and breastfeeding are among the first exclusion criteria for clinical trials and pharmacokinetic studies, resulting in significant knowledge gaps about medication safety and efficacy for these populations [4]. Worldwide it is estimated that more than half of all women require some form of medication during breastfeeding [5] yet there is an incomplete understanding of how much drug may reach the breastfed infant and what its effects may be. Pharmacokinetic studies explore how a drug passes through the body including how much enters different compartments such as the breast. This approach can support dose prediction and treatment allocation for specific groups like pregnant and breastfeeding women. Regulatory authorities use this data to assess drug safety, efficacy, and quality [6].

Recently, there has been a paradigm shift in research practices to prioritize the fair inclusion of pregnant women in research. This acknowledges the importance of including these women in research because it is the right thing to do ethically, and scientifically, and for society to ensure accurate drug dosing, improve treatment outcomes, reduce off-label drug use thus reducing chances of harm, and fill critical knowledge gaps among the population [1]. Lyerly et al. advocate for “*fair* inclusion” of pregnant women in research rather than systematic exclusion which is considered unfair or unjust because it has resulted in a significant lack of data on how medications and treatments affect pregnant women and their fetuses [7], in comparison to other patient groups. The benefits of pregnant and breastfeeding women participating in research extend beyond individual health to encompass scientific progress, improved healthcare quality, and societal well-being [8]. This lack of specific data means that frequently medication is prescribed ‘off label’, without relevant specific information. In April 2024 the US Joint National Academies released a report showing that whilst liability concerns have often been raised with respect to research in pregnancy and lactation, no such cases have occurred since 1962 when the FDA began requiring trials prior to drug marketing [9]. This report concludes with nine specific recommendations to safely increase research participation and address the critical knowledge gap.

Pharmacokinetic studies in pregnancy and lactation are frequently conducted post-marketing, but the number of these studies remains very low [10, 11]. Few studies have investigated barriers to study participation [12]. Researchers from high and middle-income countries have reported challenges in their efforts to recruit participants of this demographic, including, obstacles to study participation, lack of planning and implementing effective recruitment strategies, and costs of the recruitment phase of clinical trials [13]. In many cases, ‘ethical and logistical constraints’ are cited as a reason not to sample from the breastfed infant, without providing further details on the nature of these constraints. Over the past ten years, our team has built expertise in studying pharmacokinetics in pregnancy and breastfeeding at the Infectious Diseases Institute, Makerere University (IDI). In contrast to many other reported studies, we have achieved full recruitment and an extremely low loss to follow-up rate in all studies, and in no situations have we encountered challenges in obtaining blood samples from the breastfed infant. This paper explores our experiences, challenges, and best practices in achieving this, focusing on five recent pharmacokinetic studies [14–18] in Table 1. Whilst some of these approaches could be considered ‘best practice’ in many other areas of research, these have been of particular importance in establishing trust and credibility with our local populations and building partnerships to explore these important, but critically sensitive areas of research.

**Table 1:** Eligibility studies actively involved in the recruitment of pregnant and breastfeeding women at the IDI clinic in Uganda

Study	Study design	Primary objectives	Pharmacokinetic sampling schedule	Sample size	Year
Pharmacokinetics of drugs used to treat drug-sensitive tuberculosis in breastfeeding mother-infant pairs [15].	Observational pharmacokinetic study	To define the transfer of isoniazid, rifampicin, pyrazinamide, and ethambutol to a breastfed infant. To determine the AUC, clearance, and volume of distribution of these drugs.	• Maternal blood and breastmilk collected at 2, 4, 6, 8, and ideally* 24 h post-dose. • Infant blood sample collected at maternal trough (pre-dose) and anytime for 3–8 h post maternal dose.	20 mother-infant pairs	2021-Ongoing
Pharmacokinetics of drugs used to treat uncomplicated malaria in breastfeeding mother-infant pairs [14].	Observational pharmacokinetic study	To define the transfer of lumefantrine and its active metabolite, dibutyl-lumefantrine from mother to breast-fed infant. To determine the clearance, AUC, and volume of distribution of lumefantrine and disbutyl-lumefantrine	• Maternal blood and breastmilk were collected at pre-dose 2-, 4-, 6- and 8- hours post-dose. • Breastmilk collected at pre-dose 2, 4, 6, 8 h post-dose. • In addition, sparse sampling after the third or fourth dose and lastly at 5, 7, and up to 14 days after the first dose. • Infant blood (heel prick) was collected at the maternal trough and at a random time point over the 8-hour pharmacokinetic sampling.	30 mother-infant pairs	2022-Ongoing
Investigation of the pharmacokinetics of atazanavir in pregnant women, individuals at extremes of BMI, children, and adolescents. An observational study nested within the virtual consortium. (NCT03923231)	Observational study	To describe the pharmacokinetic parameters of atazanavir currently used in the clinical care of pregnant women living with HIV and postpartum women, children and adolescents, and individuals with obesity or malnutrition. To compare these parameters to those observed in non-pregnant adults living with HIV on second-line ART who enter a dose escalation study of ATV/r + RIF	• 20 sparse pharmacokinetic profiles in each of the following groups (pregnant, child < 5, child 6–11, adolescent 12–18, obese (BMI > 30 Kg/m^2^), malnourished (BMI < 18.5 Kg/m^2^). Everyone contributes 4 samples per PK visit. • Blood Sampling at pre-dose, 2, 4, and 6* hours post-dose *Given the potential challenges of sampling in lactating mother-infant pairs, the 6-hour time point was optional	20–40 Pregnant women and children less than 5 years old.	2017–2019
Understanding the pharmacokinetics of antiretroviral drugs in breastfeeding mother-infant pairs [19].	Observational pharmacokinetic study	To determine the AUC, clearance, and volume of distribution of (efavirenz/tenofovir + Emtricitabine or lamivudine) in maternal plasma and breast milk, and upper and lower estimates of concentration in breastfed infants. To examine the impact of covariates on maternal and infant drug exposure	Intensive sampling schedule on 40 mother-infant pairs Maternal sampling: • Morning dose: 0, 1, 2, 4 and 8 h postdose • Evening dose: 12, 16- and 20 h post-dose Infant Sampling: pre-feed at the maternal trough; 2 h after a mandated feed at 2 h post-dose. sample taken only in the infants of mothers who take their drugs in the morning), at a later randomized time point (5–8 h). Sparse sampling schedule of single time point maternal plasma, breastmilk, and infant blood samples on 160 mother-infant pairs.	200 mothers infant pairs	2016–2018
Development and validation of an assay for quantitation of antiretrovirals in human breastmilk [19].	Observational pharmacokinetic study – pilot feasibility and acceptability, together with assay development	To determine the AUC, clearance, and volume of distribution of (efavirenz/tenofovir + Emtricitabine or lamivudine) in maternal plasma and breast milk, and upper and lower estimates of concentration in breastfed infants	Maternal sampling: • Morning dose: 0, 1-, 2-, 4- and 8 h post-dose • Evening dose: 12, 16- and 20 h post-dose Infant Sampling: pre-feed at the maternal trough; 2 h after a mandated feed at 2 h post-dose. the sample was taken only in the infants of mothers who take their drugs in the morning), at a later randomized time point (5–8 h).	30 mother-infant pairs	2014–2016

AUC; Area under the concentration-time curve, ARV; Anti-retroviral therapy, ATV/r; ritonavir-boosted atazanavir, RIF; Rifampicin, AUC; Area under the curve, BMI; Body mass Index

### Strategies to promote recruitment of pregnant and breastfeeding women for pharmacokinetic studies

Across our research portfolio, several strategies have been employed to effectively recruit participants. Alongside our research, a dedicated community engagement and involvement theme has enabled a multifaced approach. The ATtaining EQUity of Access TO Research (At The EQUATOR) program [20], has the aim to transform the research culture by ensuring equity of access to research. This ‘access’ includes both equitable opportunity to participate in research, and also equity in being able to access and understand the findings of such research. A dedicated public engagement officer (SPA) ensures clear, ongoing dialogue. This is important in all settings but has particular value in the diverse, multicultural population of Uganda where there are 56 tribes and 40 spoken languages, together with highly variable socio-economic and educational status.

### Community advisory board meetings

Community advisory boards (CAB) serve as a voice for the community and study participants and provide essential input into study design and local study procedures. CABs work to ensure that research strategies acknowledge and respect the values and cultural/ethnic differences among participants. At the IDI, CAB membership includes community members who share characteristics of study participants, study participants themselves, representatives of organizations working with HIV-related programs, religious or community leaders, representatives of persons with disability, and professionals with relevant research and/or scientific expertise. The Maternal Infant and Lactation pharmacokinetic studies (MILK) hold CAB meetings quarterly to foster relationships and trust with the community. These meetings enable full discussion of protocols, and community feedback that is incorporated into the protocol and informed consent forms (ICFs) before initial regulatory ethics submissions [14]. The CAB plays a key role in creating culturally sensitive and easily understandable informed consent materials, which are essential to ensure that participants fully comprehend what they are agreeing to.

### Inclusion of peer mothers as co-investigators

Patients and research participants often feel more able to fully explore their concerns with an individual they can easily relate to. For many years, IDI has employed ‘peer mothers’ as volunteers to support high-quality delivery of clinical services; This approach has been particularly valuable in addressing conditions associated with stigma, such as HIV and tuberculosis. The MILK team includes a peer mother (JK) as a co-investigator, enabling participatory research. The involvement of peer mothers and CAB members from the early stages of protocol development has ensured that the study protocols address specific concerns and cultural contexts relevant to pregnant and breastfeeding women, thereby enhancing the ethical and cultural sensitivity of the research.

### Establishing credibility and trust through community engagement

In Uganda, there is a strong cultural emphasis on protecting women and their unborn children. Some communities believe that any risk, no matter how small, should be avoided. This protective attitude can lead to reluctance to allow pregnant or breastfeeding women to participate in research, as families and communities may fear that research could harm the mother or child. Through community engagements including music, drama, and dance; interactive research prioritization workshops; radio broadcasts and co-creation of appropriate community-facing materials, the team builds credibility and trust that can help alleviate fears and misconceptions.

In addition to communities, it has been necessary to establish trust and credibility with clinical teams at the local health centers. explored diverse ways to reach out and establish strong collaborations with community clinics that provide initial care to women; members of these teams have contributed valuably to study recruitment by referring potential participants. During community engagements with the clinical teams at these sites, we provide information, education, and communication (IEC) materials to create further awareness about pharmacokinetic studies which has been beneficial to the potential participants, their families and communities, and the clinical teams at the sites. Among the materials distributed are posters, flyers, T-shirts, booklets, and leaflets. Flyers and posters are regularly distributed at study recruitment sites; hospitals, patient noticeboards, and clinician rooms with contact details of the study teams for interested individuals who may wish to acquire more information about the studies. The tools have an educational role in raising the awareness of existing services as has been reported by other investigators [21]. Samples of the IEC materials can be assessed at; https://drive.google.com/file/d/1EyQw3MlZPTkBEgubxHEn5Aa1QYbxpy19.

### Established recruitment sites

At each collaborating site, two designated staff members; the medical officer or clinical officer and a nurse are tasked with facilitating participant recruitment and liaising with the study team. These staff members receive thorough training on the study protocol and participate in ongoing medical education sessions to ensure a comprehensive understanding of the study recruitment procedures. To support effective recruitment, the study team conducts monthly face-to-face meetings with these recruitment contacts to review the inclusion criteria, monitor recruitment progress, address recruitment challenges, and develop strategies to overcome any obstacles. These meetings also provide an opportunity for continuous support and problem-solving, ensuring that recruitment efforts remain aligned with the study’s objectives.

### Safety protocols and ethical considerations

The studies mentioned are observational pharmacokinetic studies and thus no interventions from us and therefore minimal risk to participating in our studies. In these, the decision to start treatment has already been made by the clinician responsible for the participant. Investigation of pharmacokinetics requires lengthy visits to the clinical trials unit with a sampling of the mother’s blood, breast milk, and infant blood. Concerns cited by other investigators include the length of the study visit, the acceptability of sampling breast milk, and the willingness of the mother for the infant to undergo a blood test. As with any clinical study, these pharmacokinetic studies implement rigorous safety protocols that undergo ethical review by both international and local regulatory committees. We have trained seasoned nurses/doctors who are well-versed in risk assessment, who are skilled at taking samples, and who are trained to communicate well and give participants reassurance and feedback. Also, the studies have standard operating procedures (SOPs) specific to this population.

### Stakeholder meetings

The studies have engaged policy-making stakeholders to enhance the relevance, quality, and impact of our studies while fostering collaborative partnerships that benefit all involved parties. Engaging with entities including the National Drug Authority (NDA), Ministry of Health (MOH), and Uganda National Council of Science and Technology (UNCST) helps improve the overall research landscape. Additionally, the UNCST has a desire to include adolescent pregnant and breastfeeding women in studies because they are keen that our research reflects our population where a quarter of women have their first child before age 18.

### Use of diverse communication platforms and social media

A comprehensive range of potential audience groups is targeted, each with tailored communication activities and approaches including the use of social media platforms like Facebook, YouTube, Twitter, LinkedIn, Instagram, and the mainstream media. Examples of our content can be viewed at; https://www.youtube.com/@attheequatorug/videos. Social media is an internet-based form of communication. The utilization of social media is on the rise in Uganda, with approximately 49.1% ownership of mobile phones, 25.5% internet usage, and 23.4% utilization of social media platforms [22]. The content is translated into the most spoken language in Uganda for easier communication with the study and potential participants. The study’s communications and content materials are delivered in appropriate local languages. Increasingly, we use a participatory approach to outreach whereby discussion on research and the emerging results is led by community representatives including religious leaders, as well as pregnant and breastfeeding women with disabilities.

### Barriers encountered during recruitment

#### Failure to come to the recruitment site

Enrolment of breastfeeding mother-infant pairs, particularly when the mother is clinically unwell, is challenging. An illustrative example is a protocol [14] that requires breastfeeding women diagnosed with malaria to visit the study site before receiving their initial dose of artemether/lumefantrine. Despite the efforts of research staff to reassure potential participants that enrolling in the PK study does not preclude their right to continue seeking healthcare at their primary facility, many individuals still express concerns about relocating to the PK site.

#### Change of staff in recruitment healthcare sites

Research protocols face significant disruption when the local clinic team members who have been sensitized to the importance of research in this population and trained in pharmacokinetic procedures are transferred to different sites or new roles, a situation that occurs frequently in the government healthcare system. When experienced staff members leave, there is a risk of losing critical, specialized knowledge essential for recruiting this demographic effectively. New staff receive comprehensive training on pharmacokinetic protocols, ethical considerations, and recruitment methods specific to pregnant and lactating women. However, the time needed to familiarize them with these aspects can cause delays in recruitment. This may ultimately reduce the effectiveness of participant engagement.

### Recommendations for recruitment of pregnant and breastfeeding women

**Fig. 1:**
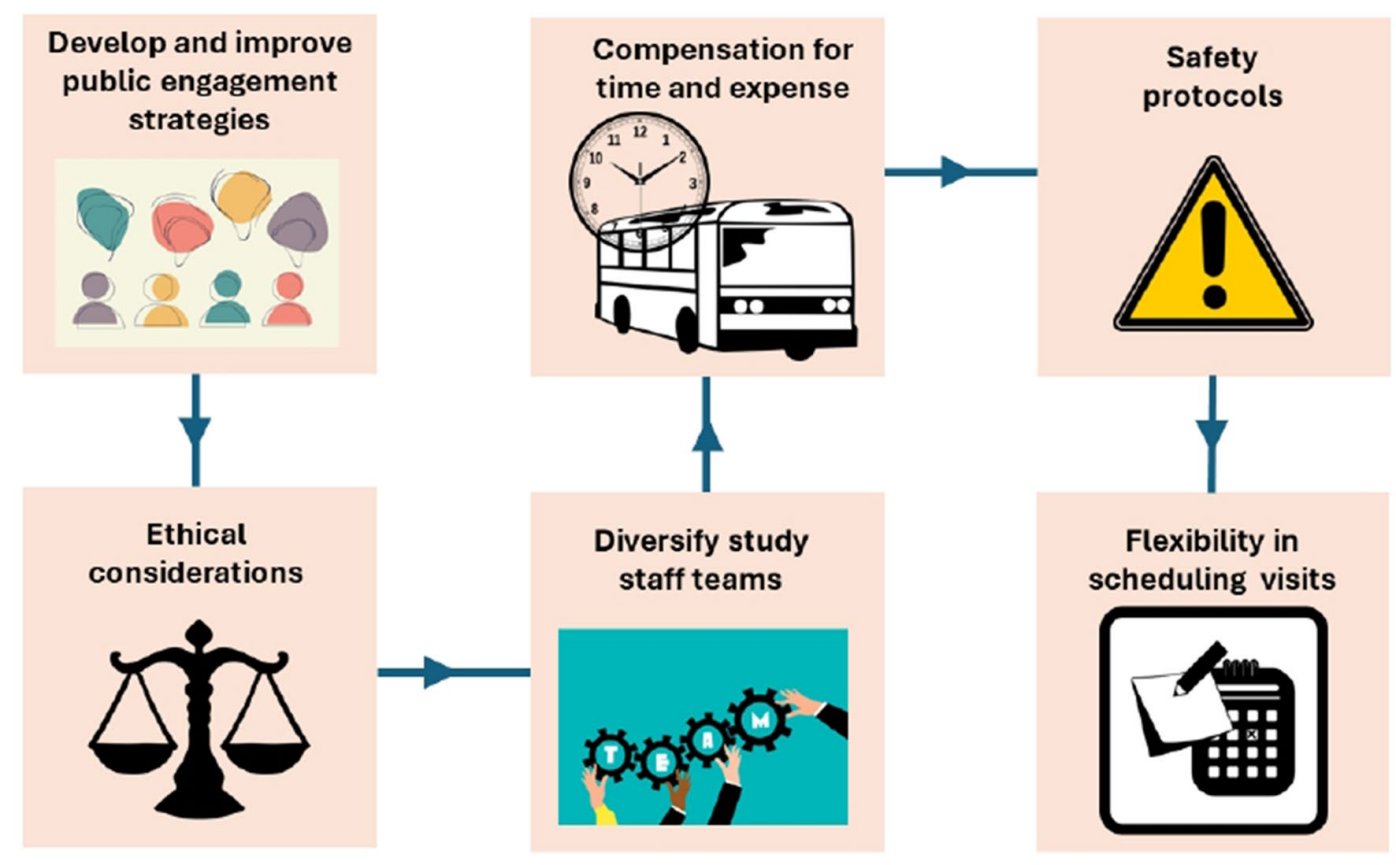
Recommendations for recruitment of pregnant and breastfeeding women in pharmacokinetic studies

Alongside the report on advancing clinical research with pregnant and lactating populations [23], we provide insights into ethically and efficiently recruiting pregnant and breastfeeding women for pharmacokinetic studies. This encompasses engaging with the public actively to increase awareness and build trust through culturally appropriate messaging and materials targeting the population. Offering flexibility in research visits to accommodate the needs of breastfeeding women, such as varied scheduling options and locations, enhances accessibility while upholding ethical principles of justice, doing no harm, respect, and beneficence to ensure women’s safety, and inclusivity [1]. Collaborative community engagement and sustained follow-up mechanisms also boost participant engagement, enriching the validity and significance of research findings, as outlined in Fig. 1 which illustrates recommendations for recruitment in the study population.

## Conclusion

Potential safety risks and ethical concerns in pharmacokinetic research involving pregnant and breastfeeding women can be addressed through several key measures. Implementing robust safety protocols with comprehensive risk assessments and monitoring procedures ensures the protection of both mother and infant. Providing clear, culturally sensitive informed consent processes that help participants fully understand the research and its potential risks. Engaging patient advocates and community representatives in protocol development can address specific concerns and enhance ethical sensitivity. The incorporation of peer mothers and public engagement officers as co-investigators ensures seamless dialogue with diverse communities and rapid responses to emerging concerns. Regular training for research staff and ongoing ethical oversight further ensure adherence to high standards of care and support, thus mitigating risks and upholding ethical principles throughout the study. The commentary contributes to the advancement of pharmacokinetic research in marginalized groups and underscores the importance of inclusivity and support in clinical research endeavors.

## Data Availability

https://drive.google.com/file/d/1EyQw3MlZPTkBEgubxHEn5Aa1QYbxpy19 https://www.youtube.com/@attheequatorug/videos
